# Mechanisms of Change Underlying Effects of an Early Parenting Intervention on Child Development Among Vulnerable Families in Rwanda

**DOI:** 10.3390/children13030344

**Published:** 2026-02-27

**Authors:** Sarah K. G. Jensen, Matias Placencio-Castro, Shauna M. Murray, Vincent Sezibera, Theresa S. Betancourt

**Affiliations:** 1 Women and Infants Hospital, Providence, RI 02905, USA; 2 School of Social Work, Boston College, Chestnut Hill, MA 02467, USA; placenci@bc.edu (M.P.-C.); theresa.betancourt@bc.edu (T.S.B.); 3 Lynch School of Education and Human Development, Boston College, Chestnut Hill, MA 02467, USA; 4 School for Global Inclusion & Social Development, University of Massachusetts, Boston, MA 02125, USA; shauna.murray@umb.edu; 5 Center for Mental Health, University of Rwanda, Kigali P.O. Box 3286, Rwanda; v.sezibera@ur.ac.rw

**Keywords:** parenting, intervention, mechanisms, theory of change

## Abstract

**Highlights:**

**What are the main findings?**

- By empirically testing the Theory of Change underlying a home-based parenting intervention, we show that the effects of a parenting intervention on children’s developmental outcomes among vulnerable families in rural Rwanda are explained by key changes in caregivers’ behaviors to improve the home environment and child nutrition.
- The Sugira Muryango parenting intervention successfully improved caregiving behaviors related to stimulation, play, and early language learning, which in turn were associated with improvements in child development.

**What are the implications of the main findings?**

- By understanding how interventions work, researchers can continuously refine interventions to better support families and early childhood development.
- By examining the Theory of Change, we were able to identify strengths (e.g., improved home environment and nutrition) and areas of improvement (e.g., identifying ways to link improved caregiver emotion regulation and violence reduction to better child development outcomes).

**Abstract:**

Background: Intervention effectiveness studies rarely empirically assess Theories of Change (ToC) to determine how an intervention worked. We examine the ToC underlying the Sugira Muryango (SM) parenting program in rural Rwanda to understand whether the intervention improved child development outcomes via changes in caregivers’ behaviors to improve the home caregiving environment, as hypothesized. Methods: SM uses coaching of parents to create a safe, affectionate, stimulating, and violence-free home environment. A cluster randomized trial enrolled 1049 families with young children. SM had immediate effects on caregiver behaviors, improving scores on the Home Observation for Measurement of the Environment (HOME), harsh discipline, caregiver emotion regulation, and provision of dietary diversity. We use structural equation modeling to examine whether change in caregivers’ behaviors explains intervention-related improvements in child development (Ages and Stages Questionnaire) one year after the intervention ended. Results: Improvements in positive caregiving practices, including stimulation and early language learning as captured by the HOME, explained some of the intervention-related changes in child development, including gross motor, communication, problem-solving, and personal-social development. Increased dietary diversity explained intervention-related change in gross motor, problem-solving, and personal-social development. Change in harsh discipline and caregiver emotion regulation did not explain child outcomes. Conclusions: Intervention-related changes related to constructs captured on the HOME and dietary diversity were associated with changes in child development scores, but violent discipline and caregiver emotion regulation were not. Future research should examine whether these components of the intervention can be strengthened and may influence child development via other pathways, for example, via caregiver mental health.

## 1. Introduction

Establishing intervention effectiveness is a crucial step in the evaluation of an intervention, but empirical testing of how an intervention works can add immense value. Such a mechanistic analysis can confirm whether a program works as intended and guide program refinements and implementation by supporting the continued improvement of program materials. A Theory of Change (ToC) is a theoretical framework often expressed in the form of a diagram that maps the pathways by which an intervention is expected to achieve its effects [1]. A ToC can thus guide the theory-based evaluation of a program [2]. A ToC breaks a complex intervention into smaller, measurable steps by linking specific objectives to the intended outcomes and mapping the specific intervention components that are intended to drive such effects. A ToC may even highlight stakeholders and projected barriers in implementation. Ideally, the development of a ToC begins in early program development; the Medical Research Council recommends that the establishment of a theoretical understanding of the process of change is essential before progressing from pilot to a full intervention effectiveness trial [3].

Empirical testing of an intervention’s ToC can reveal which components of the intervention are most influential in driving change. These highly effective components—sometimes referred to as the “active ingredients’’—may be prioritized during training and implementation to ensure high fidelity, given their importance in driving positive outcomes. Conversely, identification of “inactive” components allows researchers to either refine or remove elements that do not contribute to the intervention’s success.

**Figure 1:**
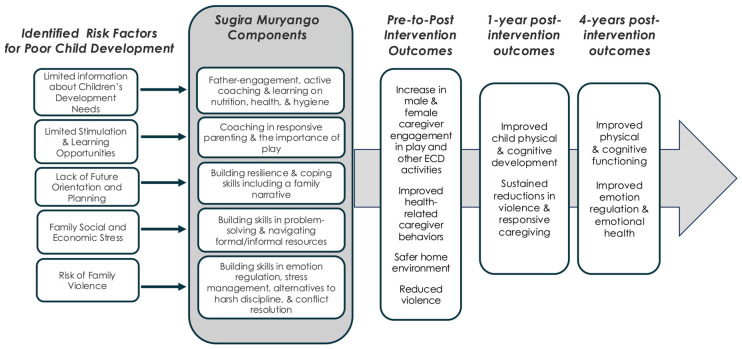
Sugira Muryango Theory of Change adapted from Jensen et al. 2025 [6].

Here, we examine the ToC underlying the Family Strengthening Intervention for Early Childhood Development and Violence Prevention (FSI ECD + VP or “Sugira Muryango”) as implemented in a cluster randomized trial (CRT) in Rwanda [4]. Sugira Muryango (SM) (“*strong family*”) is a lay-worker-delivered, home-visiting parenting intervention that uses coaching to support male and female caregivers of young children living in extreme poverty and who often experience compound adversity. The SM ToC shown in Figure 1 identifies family-level risk factors, such as poor caregiver mental health, familial violence, and limited developmental knowledge, and links them to specific intervention components designed to optimize the child’s early care environment. The ToC is grounded in early formative work conducted to map early child development (ECD) needs in Rwanda [5]. While that work initially informed the design of community-based ECD centers that provide play-based learning and nutrition counseling, a key service gap remained: the ECD centers only serve children aged 3–6. SM was designed to fill this gap by offering home-based parenting services to families with children who are too young to be eligible for center-based care but are at risk for underdevelopment due to extreme poverty.

SM leverages coaching and education to generate behavior changes in all caregivers supporting the care of the targeted child, including fathers. The curriculum covers nutrition, health, hygiene, responsive play, and non-violent discipline. Additionally, to address common stressors among families living in severe poverty and post-conflict environments, the program helps caregivers navigate available resources and practice skills for stress reduction, emotion regulation, and conflict resolution.

As shown in previously published effectiveness studies, SM led to immediate positive change in caregiver behaviors, increasing caregivers’ engagement in responsive play interactions, reducing the use of harsh discipline, decreasing victimization of women to intimate partner violence, and increasing caregivers’ reports of providing dietary diversity and improved hygiene [4]. One year after the intervention ended, most of these caregiver behavior effects were sustained, and children whose families received SM demonstrated greater gains in developmental milestones across domains of gross motor, communication, problem-solving, and personal-social development compared with the children whose families received usual care. This current paper extends prior effectiveness findings by exploring the hypotheses underlying the SM ToC, namely that immediate changes in specific caregiver behaviors led to the observed improvements in children’s development outcomes observed one year later. By identifying caregiver behaviors associated with improved child outcomes, we continue to refine the SM program during the continued rollout and expansion to other settings.

## 2. Methods

### 2.1. Study Design

The SM cluster-randomized trial (CRT) was implemented in Rwanda between 2018 and 2019. Three districts (Ngoma, Nyanza, and Rubavu) were purposively selected because they were identified by the Government of Rwanda to have full operational rollout of the Government of Rwanda’s flagship social protection program, the Vision 2020 Umurenge Programme (VUP), including a “classic Public Works” (cPW) and “expanded Public Works” (ePW) program by the time of the CRT. cPW provides seasonal, labor-intensive infrastructure employment to labor-endowed poor households with only one work-able caregiver, while ePW offers more flexible, year-round community-based work opportunities for households with partial labor capacity and caregiving responsibilities. Ensuring that both modalities were fully implemented in study districts was a core eligibility criterion, as it allowed the trial to enroll households across the full spectrum of VUP public works beneficiaries and ensured consistent exposure to the national social protection platform. Eligible households were identified from government administrative lists of families classified as Ubudehe 1 and enrolled in VUP. Additional inclusion criteria were: (1) eligibility for VUP participation; (2) presence of at least one child aged 6–36 months; and (3) willingness to participate in a home-visiting intervention. A total of 1049 households were enrolled between February and April 2018. Baseline, post-intervention, and 1-year follow-up data collection were conducted by Laterite Rwanda, a Kigali-based research firm, with fieldwork for the follow-up implemented between August and September 2019 using trained local enumerator teams operating under standardized protocols and field supervision.

### 2.2. Procedures and Randomization

Non-overlapping, geographically defined clusters were created by combining one or more contiguous villages to facilitate intervention delivery while simultaneously ensuring enough distance between villages in different clusters to avoid potential spillover of the intervention effect, which may occur if treatment families begin to teach other families in their village about the content of the intervention. The CRT randomized families to treatment and “usual care” conditions at the cluster level with 198 clusters. *n* = 508 families were allocated to “usual care” (VUP only), while *n* = 541 were allocated to treatment (VUP + SM). Data were collected at baseline (pre-intervention), immediately after the intervention ended, and follow-up data were collected 12 months after the intervention ended. Adult caregivers were read the consent form and provided written informed consent in the form of a signature or a thumb print consenting for themselves and their child(ren) to participate. We did not track the rate of families that consented as opposed to declined participation.

### 2.3. Participants

We include data from 1049 children and their primary caregiver. The primary caregiver was defined as the caregiver who self-identified as knowing the child best, most often the biological mother. We excluded 35 children who were the second enrolled child within the same household to avoid nesting of data within households. Attrition from baseline to 12 months post-intervention was 2% for children and 10% for caregivers. Handling of missing data followed intention-to-treat protocols and is described below under statistical analysis.

Descriptive sample information is provided in Table 1. Among treatment households, 91% of the primary caregivers were mothers, 1.5% were fathers, and 7.5% were other familial caregivers such as grandparents, aunts, or uncles. Among control households, 90% of primary caregivers were mothers, 1% were fathers, and 9% were other caregivers. Levels of education were similar across the two groups, with 49% reporting some to 6 years of schooling and around 23% reporting no schooling. This level of education is lower than the average for all women in Rwanda, with 9% reported as having no education in 2019 [7], but consistent with the vulnerable status of eligible households. The average age of children in the treatment households was 21.1 months, and 48% were female. The average age of children in the control households was 22.1 months, and 54% were female. The prevalence of stunted growth among children (height-for-age < 2 SD below the WHO Child Growth Standards median) was 48% in both groups.

### 2.4. Intervention Component

The SM intervention was implemented between May and September 2018. SM consists of 12 home-based sessions lasting 60–90 min and during which all caregivers and children living in the household are encouraged to participate. Lay worker interventionists were selected using a three-step process that involved (a) nominations from community members, (b) phone screenings, and (c) in-person interviews during which candidates were asked to reflect on materials from the intervention, complete a writing test, and answer questions about prior experiences delivering community-based programs [4]. Selected interventionists then completed three weeks of intensive training. During program delivery, ongoing supervision included in-person supervision during the first 3 weeks, weekly telephone supervision, and monthly in-person group supervision. Additionally, weekly in-person peer support groups were conducted to promote fidelity to the program.

SM uses active coaching with the aid of visual booklets tailored to the Rwandan culture. Every session includes a 15 min “coached” session of active play where male and female caregivers receive real-time feedback on their interactions with their child to enhance engagement in positive and responsive interactions. Sessions are delivered at an average pace of one module per week but can be tailored to meet each family’s needs. For example, flexibility is provided to accommodate parents’ schedules and increase the participation of both male and female caregivers in all sessions. The SM curriculum builds on the Nurturing Care Framework developed by the World Health Organization, UNICEF and World Bank [8], and was further tailored to the Rwandan context. Moreover, to address the high prevalence of harsh discipline and other forms of family violence, we added content to reduce caregiver stress and interpersonal conflict. Two booster sessions were delivered three and six months after the intervention ended. These booster sessions allowed the interventionist to reconnect with the family and to identify and address ongoing challenges.

### 2.5. Outcomes

Data to support the program evaluation were collected at baseline (January 2018), immediately after the intervention (September 2018), and one year after the intervention ended (August 2019). Measurement tools to assess caregiver behaviors and child development outcomes utilized direct observation and/or caregiver interviews conducted in Kinyarwanda by trained and local enumerators hired and managed by Laterite in collaboration with researchers at Boston College and the University of Rwanda. Caregiver interviews were conducted at the household or other private locations. Assessments of children were done in private rooms at central locations such as cell and sector offices or churches. Assessors were blinded to families’ intervention status at all timepoints.

Home Environment: The home environment was assessed using the Home Observation for Measurement of the Environment (HOME) [9], which uses a blend of observation and caregiver report. We used a slightly adapted 43-item version previously used in Uganda [10]. Items are coded as binary (yes/no) and cover different domains of the child’s home environment, including social interactions (e.g., the caregiver responds verbally to the child’s talk), caregiver expressions of warmth (e.g., the caregiver caresses, strokes head, or kisses the child), and negative interactions (e.g., caregiver shouts at child). Parent-reported items include recent engagement in stimulating activities with the child (e.g., talking to the child while doing chores; reading and telling stories to the child; teaching the child new things), exposing the child to other people and environments (e.g., playmates and new places) and access to learning materials (e.g., books, manufactured toys, and homemade toys). A total HOME score was created by summing all items. Higher scores reflect environments that promote child wellbeing and learning (Maximum 43; Cronbach’s α = 0.76).

Violent discipline: Violent discipline was assessed using items from UNICEF’s MICS Child Discipline Module (UNICEF, New York, NY, USA) [11]. Caregivers reported on their engagement in the following behaviors within the past 30 days as “yes/no”: shouting at the child, screaming at the child, calling the child demeaning names, shaking the child, spanking the child, slapping the child, or beating the child. We counted the number of “yes” responses to create a total score (Range 0–7).

Caregiver emotion dysregulation: Caregiver emotion dysregulation was assessed using an abbreviated 24-item version of the Difficulties in Emotion Regulation Scale (DERS) (NovoPsych, Melbourne, VIC, Australia) [12], described in detail elsewhere [13]. The DERS assesses difficulties in emotion regulation related to poor awareness of emotions, poor acceptance of emotions, difficulties engaging in goal-directed behavior, difficulties refraining from impulsive behavior when experiencing negative emotions, and poor access to emotion regulation strategies. The response scale indicates how frequently an item applies on a five-point scale ranging from ‘almost never’ to ‘almost always’. Items were summed to create a cumulative score (Maximum 96; Cronbach’s α = 0.95).

Dietary Diversity: Child dietary diversity was assessed using household food access items developed by USAID [14]. The dietary diversity score reflects how many of the seven food groups (grains, roots, and tubers; legumes and nuts; dairy products; meat, fish, poultry, and organ meats; eggs; fruits and vegetables rich in vitamin A; other fruits and vegetables) the child consumed in the past 24 h (Maximum 7).

Child development: Child development was assessed using the Ages and Stages Questionnaire (ASQ) version 3 [15]. The ASQ uses a series of age-specific parent questionnaires to assess the developmental stage of the child and screen for developmental delay of children in the areas of gross motor, fine motor, communication, problem-solving, and personal-social development. We used continuous total raw scores since the standardized scores and cut-offs for developmental delay have not been validated for use in Rwanda. We excluded fine motor outcomes as previous work found no intervention effects on fine motor.

### 2.6. Statistical Analysis

#### 2.6.1. The SEM Model

**Figure 2:**
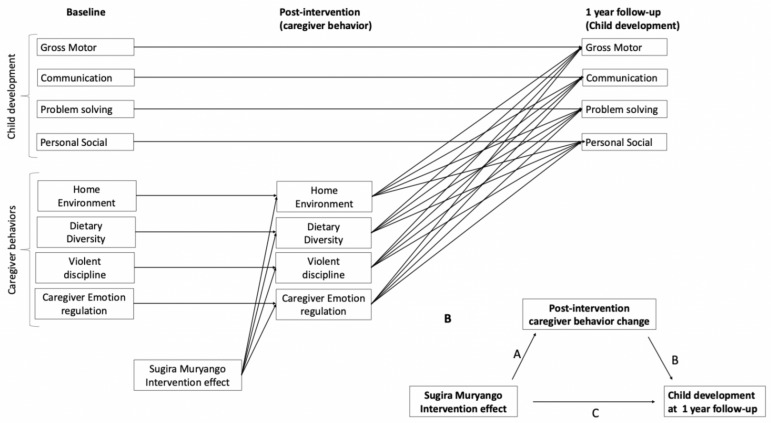
Structural Equation Model examining mechanisms of change related to the Sugira Muryango intervention’s effects on child outcomes.

The hypotheses stated in the SM ToC were examined within a structural equation model (SEM) as outlined in Figure 2. The SEM model includes auto-regressive paths that connect repeated variables across timepoints (illustrated as flat lines) and cross-lagged paths that connect different variables across timepoints (illustrated as sloped lines).

The autoregressive paths were included to account for time dependence across measurement waves in caregiver behaviors (baseline to post-intervention) and children’s development outcomes (baseline to 12-month post-intervention scores) and to examine effects of the intervention on change in caregiver behaviors (baseline to post-intervention) and change in child outcomes (baseline to one-year follow-up).

We included the cross-lagged paths from treatment status to caregiver post-intervention behavior scores to determine how much of the observed caregiver changes in caregiver behaviors could be explained by the SM intervention. Although the ToC states that changes in child outcomes are driven by the indirect paths via caregiver behaviors on children’s outcomes as opposed to by the intervention itself, we included direct cross-lagged path from intervention status to child outcomes at the one year follow up because this allows us to estimate the residual effect of the intervention while accounting for any potential intervention effects that could act as confounders when explaining group differences in child development outcomes via the indirect pathways. As a holistic intervention, SM encompassed components beyond those included in the statistical model, such as behaviors of other caregivers in the home, strategies for navigating formal support networks, family functioning, and supporting children’s health. Omitting direct paths of the intervention on children’s outcomes could bias the estimates of indirect effects, given that the influence of intervention components not modeled as mediators would remain unaccounted for, thus acting as unmeasured confounders. Finally, we estimated the cross-lagged effects of caregiver behaviors at post-intervention (corrected for baseline scores via the autoregressive paths) on child development scores (corrected for baseline scores via the autoregressive paths). Based on the ToC, we included the following caregiver behaviors as potential mediators of changes in children’s developmental (ASQ) outcomes: home environment scores (HOME), violent discipline scores, caregiver emotion dysregulation scores, and child dietary diversity scores. We did not include the clustering variable in the model because the intraclass correlations (ICCs) were small, ranging from 0.001 to 0.117. Sensitivity analyses that included clustered estimates showed that results were not meaningfully different and are included in the Supplemental Materials. In the supplemental analyses, we also provide the estimates from one alternative model that included child development assessments at post-intervention and estimated the autoregressive paths from baseline child development scores to post-intervention and from post-intervention child development scores to the 12-month follow-up, along with the full covariance structure between all variables at post-intervention. This model would be appropriate if we believed that the intervention has direct and immediate effects on child development outcomes, but since the ToC explicitly hypothesizes that changes in child development outcomes are driven by changes in caregiver behaviors, we present a parsimonious model that best represents the ToC. We also note that estimates derived from the alternative model were not meaningfully different from the presented model, therefore not changing any of the conclusions presented here, and model fit was slightly worse than the more parsimonious model.

All the analyses were conducted in R (version 4.5.2), using Lavaan (version 0.6-20) [16]. Models were estimated via Full Information Maximum Likelihood, with bootstrapped, bias-corrected confidence intervals. Acceptable model fit was assessed using the following criteria: Comparative Fit Index (CFI) > 0.90; Standardized Root Mean Squared Residual (SRMR) < 0.08, and Root Mean Square Error of Approximation (RMSEA) < 0.06 [17,18]. We did not consider the chi-square statistics in the evaluation of model fit due to its sensitivity to sample size [19]. RMSEA values were interpreted with caution as they usually indicate better model fit with larger degrees of freedom [20]. Individual structural paths were considered significant if *p* < 0.05 and the 95% did not include zero.

#### 2.6.2. Indirect Effects

We use estimation of indirect effects to quantify how much of the change in child development scores (between baseline and the 12-month post-intervention) could be explained by changes in caregivers’ behaviors (from baseline to post-intervention). The indirect effects were defined as the product of the structural paths from the intervention to the caregiver behavior at midline (A paths) and the paths from the caregiver behavior score post-intervention to the follow-up child development outcomes (B paths). We report indirect effects on child outcomes via caregiver behaviors if two conditions were met: (1) the path from treatment status to caregiver behaviors at post-intervention is significant (significant A path) and (2) the path from caregiver behavior change to child outcomes is significant (significant B path).

## 3. Results

### 3.1. Structural Equation Model Fit

The examined model shows acceptable fit to the data: RMSEA = 0.049; CFI = 0.965; TLI = 0.937; SRMR = 0.053. Standardized regression coefficients for each path, along with exact *p*-values and 95% confidence intervals, are provided in Table 2. Standardized coefficients for indirect effects and total effects (indirect + direct effects) are provided in Table 3 and Table 4, respectively.

### 3.2. Caregiver Behaviors

As expected, we found that caregiver behaviors at baseline predicted post-intervention as observed in the significance of the auto-regressive paths (all *p* < 0.05). In line with previously published findings, we also found that treatment status predicted post-intervention caregiver outcomes as reflected in increased HOME scores (Est. = 0.391, 95% CI: 0.340 to 0.446, *p* < 0.001), decreased violent discipline (Est. = −0.16795%, CI: −0.221 to −0.115, *p* < 0.001), and increased dietary diversity (Est. 0.169 = 95% CI: 0.111 to 0.225, *p* < 0.001). We also observed only a borderline significant direct treatment effect on decreased caregiver emotional dysregulation (Est. −0.054 = 95% CI: −0.105 to 0.002, *p* = 0.046). Because of the inclusion of the auto-regressive paths from baseline to post-intervention caregiver behaviors, these results account for baseline behaviors and thus reflect treatment effects on change in caregiver behaviors from before to after the intervention.

### 3.3. Treatment Effects on Change in Child Development Outcomes

As expected, child development scores at baseline predicted one-year follow-up outcomes as shown in the significant auto-regressive paths (all *p* < 0.05). We did not see any significant paths from SM treatment status to child development outcomes. This fits our hypothesis that the direct treatment effects are driven by the mediating changes in caregiver behaviors as outlined below. The inclusion of caregiver behavior changes as predictors of child outcomes likely canceled out the treatment effects in the model since they explain the same variance as the indirect paths.

### 3.4. Indirect Effects of Treatment on Change in Child Outcomes via Caregiver Behavior Change

As mentioned above, we examined indirect effects only if the following two conditions were met: treatment status predicted changes in a caregiver’s behaviors from baseline to endline, and changes in a caregiver’s behaviors from baseline to endline predicted change in child outcome. This was true for all paths involving the home environment (HOME) and dietary diversity, but not for violent discipline and caregiver emotion regulation. Examining the indirect effects (Table 3), we found that positive change in the home environment significantly explained increases in all child outcomes, namely gross motor development (Est. = 0.070, 95% CI: 0.041–0.099, *p* < 0.001), communication (Est. = 0.102, 95% CI: 0.074–0.131 *p* < 0.001), problem-solving (Est. = 0.076, 95% CI: 0.049–0.102, *p* = 0.001), and personal-social development (Est. = 0.075, 95% CI: 0.050–0.100, *p* < 0.001). Increases in dietary diversity from baseline to post-intervention explained positive change in three of four child outcomes, namely gross motor (Est. = 0.012, 95% CI: 0.002–0.025, *p* = 0.039), problem-solving (Est. = 0.011 95% CI: 0.001–0.024, *p* = 0.047), and personal-social development (Est. = 0.013, 95% CI: 0.003–0.027, *p* = 0.024). We did not see an indirect effect of SM on child communication scores via dietary diversity.

## 4. Discussion

This study casts light on the mechanistic ways in which the FSI ECD + VP (Sugira Muryango in Rwanda) parenting intervention achieved a positive impact on children’s development. We find that positive changes in children’s development scores are explained by changes in specific aspects of change in caregivers’ nurturing care behaviors following the intervention. We hereby provide important empirical support for the SM ToC, which states that intervention effects on children are not driven by the intervention per se, but by the effects of the intervention on the child’s home environment. We further highlight specific behavior changes that drove improvements in child outcomes over time. Out of the four pathways of intermediary caregiver behavior change, we found support for two mediators of positive child outcomes, namely, a) improved home environment scores that reflect the presence of positive maternal interactions, absence of negative interactions, and presence of learning materials and b) increased dietary diversity. We did not find support for changes in caregivers’ use of violent discipline or improved caregiver emotion regulation as drivers of child-level cognitive or socio-emotional outcomes.

The ToC was modeled using an SEM framework, which allowed us to simultaneously estimate multiple sequential paths. An advantage of SEM is that it allows us to disentangle the intervention across simultaneously activated pathways to estimate the significance of each path. This allowed us to identify the key drivers of change in child outcomes and highlight successful components of the SM program. Notably, improved HOME scores predicted change in all four child outcomes and therefore emerged as the most powerful mediator of the intervention effect. The Pakistan Early Child Development Scale-Up trial (PEDS), another parenting intervention study that provided content on stimulation as well as nutrition supplementation, similarly found that change in caregiver–child interactions and child dietary diversity mediated intervention effects on child cognition measured using the Bayley Scales of Infant and Toddler Development when children were 24 months old [21], and that factors related to the home environment predicted child intelligence scores at 48 months [22]. Content to promote responsive caregiver–child interactions is central to the Nurturing Care Framework, a widely used evidence-based roadmap developed by the WHO, UNICEF, and the World Bank Group to support the healthy development of young children from conception to age three [8], and responsive care is often a central component of parenting-focused early child development (ECD) programs [23]. The importance of responsive care for supporting children in achieving developmental milestones is supported by our results that highlight the home environment as the strongest predictor of positive child outcomes. SM involved coaching of caregivers on play, stimulation, early language learning and responsive care during every single visit, and results demonstrate that this component of the intervention worked as intended. Social interactions are often the focus of ECD programs because there is a strong suggestion in the literature that caregiver interactions with children can mediate the effects of socio-economic vulnerability (i.e., poverty and low socioeconomic status) on cognitive [24] and even neurodevelopmental outcomes [25]. Indeed, a meta-analysis of 102 ECD program trials found that parenting interventions that focused on play and communication significantly improved cognitive and language outcomes in children under 3, especially in low-resource settings [23].

A novel finding of our study is that improvements in parent-reported behaviors to support children’s dietary diversity had an additional positive impact on child development outcomes. Dietary diversity has been less widely studied as a mediator of parenting program effects because many parenting programs, like SM, do not provide food or supplements, but address children’s nutrition indirectly via education and coaching. SM includes coaching on the importance of children’s diet and selection and preparation of healthy food, and our findings suggest that this component of the intervention is strong and benefited the children who received the intervention. The findings from the Pakistan Early Childhood Development Scale Up Trial (PEDS) trial [26], where dietary diversity and home environment scores similarly explained intervention effects on children’s Bayley scores at 24 months, led authors to suggest that the blended nature of the intervention, which provided both nutrition and stimulation, boosted its effectiveness [21]. Here, we extend those findings by showing that even without supplemental nutrition, which may not be accessible in many parenting programs, simple content to coach caregivers on nutrition can lead to improvements in caregiver behaviors to support a healthy, diverse diet and thereby improve child development outcomes.

Although SM was associated with reductions in violent discipline, these changes in caregiver behaviors did not predict child development outcomes. This does not align with prior studies documenting the harmful effects of violent discipline on children’s development. Working with the broader family of the child, SM introduces alternative strategies to violent discipline and addresses root causes of domestic violence, including caregiver stress, alcoholism, interparental conflict, and emotion dysregulation via caregiver coaching on emotion regulation and conflict resolution strategies and gender inequality. Current results suggest that the content of harsh discipline within SM may need further refinement to benefit child development scores. Potential changes to SM to strengthen violence reduction may involve further engagement with the community to impact social norms around harsh discipline and improve gender inequality, which is a known risk factor for violence [2]. Moreover, strategies to improve violence reduction may include increased involvement of community leaders and the use of campaigns to change attitudes. In a subsequent implementation of SM, we sought to strengthen violence prevention by training government officials and a government-appointed social protection workforce on violence prevention content from SM. Two methodological limitations may also contribute to the lack of findings regarding violence reduction. Firstly, we note that caregiver behavior change outcomes were assessed using parent self-reports, except for the HOME scale, which combines caregiver self-reports and observations. Because caregivers likely know that harsh discipline represents a socially undesirable behavior, reports may have been biased, thus limiting our ability to detect associations. Moreover, a disadvantage of examining change within a complex SEM model is that complex models can mask relationships among highly correlated variables. In this model, we did not find support for reduced harsh discipline or improved caregiver emotion regulation as drivers of child outcomes, yet we note that the interrelated nature of the examined pathways could explain the absence of effects of reduced harsh discipline and improved emotion regulation. For example, positive change in the home environment, a score that encompasses aspects of positive and negative caregiver interactions, may correlate with our measure of reductions in harsh discipline, since caregivers who are more sensitive to their child and engage in more frequent positive interactions may use less violent strategies to discipline their children.

Previous research consistently finds that corporal punishment and other forms of harsh discipline, including psychological aggression in childhood, are associated with a range of poor child outcomes, including lower cognitive scores and behavioral problems such as antisocial behavior, and externalizing and internalizing problems [27,28]. Although the use of harsh discipline is found to be more widespread in socioeconomically vulnerable households, compelling evidence suggests that effects of violence on child outcomes are independent of family-level socio-economic status [27] and country-level wealth [29]. Together, this suggests that violent discipline may be compounded by poverty but that the potential harmful effect of violent discipline on children transcends the stress of childhood poverty. Harsh discipline is common in many parts of the world, and incorporation of violence reduction into ECD programs that target socioeconomically vulnerable households should remain a priority [30]. A recent analysis of data from UNICEF’s MICS data from 49 low- and middle-income countries found that more than 60% of the world’s two- to four-year-old children had experienced aggressive physical and psychological discipline, and geographical patterns also suggested that violent discipline is most widespread in poor regions, and the highest prevalence rates were observed in South Asia and Sub-Saharan Africa [31]. Considering the continued widespread use of harsh discipline and the known impact of violence on children, ECD interventions should continue to integrate violence reduction [30,32]. Other examples of recent ECD programs that include violence reduction include the Parenting for Lifelong Health program, which has been implemented across different settings and age groups from infants through age 17 [32,33], and the ACT Action Program—Raising Safe Kids [34]. Failure of ECD programs to address child discipline or other forms of domestic violence is a lost opportunity because positive and negative caregiver interaction with children is intricately intertwined.

Key strengths of this study include the large sample size and availability of repeated measures across three waves of data collection, which positions us to conduct complex analyses to understand intervention-related mechanisms of change. Yet, for the sake of statistical parsimony, we modeled the ToC based on only four pathways. Other potential mediators include other health-promoting behaviors addressed in SM related to hygiene, intimate partner violence, and the family’s utilization of external resources that we did not examine here. We also note that this paper focused on behavior change in the primary caregiver. Since only 13 of the included 1049 families had a father classified as the primary caregiver, this inadvertently meant that the study mostly focused on behavior change in the mother. Future analyses may examine changes in caregiver behaviors while accounting for cohabitating caregivers and male caregivers. Additional limitations to the paper and conclusion that can be drawn include our reliance on self-report measures of parenting behaviors and parent-reported child assessments. Moreover, while SEM presents a suitable framework for examination of sequential effects, such as the effect of an intervention on caregiver behaviors post-intervention, on child outcomes, our inability to directly manipulate the proposed mediators outside of the broader effect of the intervention limits our ability to make any conclusions regarding causality. Finally, a limitation is the lack of data regarding the number of families that declined participation. Consequently, we cannot calculate a formal participation rate. This introduces the possibility of selection bias, as families who consented may differ systematically from those who declined, potentially limiting the generalizability of the findings to the broader population

## 5. Conclusions

Understanding the active ingredients of an intervention facilitates continuous improvement of interventions and can guide the development of briefer and more cost-effective interventions that focus on the components that most effectively facilitate positive change. This study adds to the existing ECD literature by examining the mechanistic pathways through which intervention-related changes in caregivers’ behavior positively impacted children’s development in vulnerable households in a low-resource environment in Rwanda. As hypothesized, we found that mechanistic pathways involving positive changes in the home environment, as well as a pathway related to nutritional diversity, had positive impacts on subsequent child development scores. These findings support the notion of added benefits of holistic child development interventions that coach parents to address nurturing parent–child interactions, play, stimulation, dietary diversity and health-related aspects of children’s environment to optimize children’s developmental opportunities.

## Figures and Tables

**Table 1:** Sample descriptive information.

	Control	Treatment	Total
Primary Caregivers	496	527	1023
Age in years, Mean (SD)	34.2 (9.7)	33.2 (9.1)	33.7 (9.4)
Relationship to child %			
Mother	89.57	90.94	90.28
Father	0.98	1.48	1.24
Other	9.45	7.58	8.48
Single caregiver (%)	50.00	47.13	48.52
Education (%)			
No schooling or do not know	23.23	22.18	22.69
<6 years	47.83	50.65	49.29
>6 years	28.94	27.17	28.03
Children			
Age in months, Mean (SD)	22.1 (8.5)	21.1 (8.1)	21.6 (8.3)
Gender (% Female)	53.94	47.69	50.71
Prevalence of stunting (%)	48.03	48.24	48.14

**Table 2:** Structural Equation Model results.

Outcome	Predictor	STDX Coef.	*p*-Value	95% CI LB	95% CI UB
Auto-regressive path child development baseline to 1-year follow-up					
Gross motor 2	Gross motor 0	0.153	<0.001	0.092	0.206
Communication 2	Communication 0	0.137	<0.001	0.087	0.187
Problem Solving 2	Problem Solving 0	0.141	<0.001	0.088	0.198
Personal Social 2	Personal Social 0	0.169	<0.001	0.115	0.214
Auto-regressive path caregiver behavior baseline to post-intervention					
HOME Score 1	HOME Score 0	0.283	<0.001	0.229	0.338
Dietary diversity 1	Dietary diversity 0	0.252	<0.001	0.191	0.312
Violent discipline 1	Violent discipline 0	0.506	<0.001	0.432	0.583
Caregiver DERS 1	Caregiver DERS 0	0.515	<0.001	0.449	0.576
Treatment effects on post-intervention caregiver behavior					
HOME Score 1	Treatment	0.391	<0.001	0.340	0.446
Dietary div. 1	Treatment	0.169	<0.001	0.111	0.225
Violent dis. 1	Treatment	−0.167	<0.001	−0.221	−0.115
Caregiver DERS 1	Treatment	−0.054	0.046	−0.105	0.002
Treatment effects on endline child development outcomes					
Gross Motor	Treatment	−0.014	0.670	−0.079	0.054
Communication	Treatment	−0.029	0.372	−0.091	0.038
Problem Solving	Treatment	−0.005	0.884	−0.070	0.060
Personal Social	Treatment	−0.060	0.059	−0.120	0.000
Effects of post-intervention caregiver behaviors on endline child development outcomes					
Gross motor	HOME Score 1	0.178	<0.001	0.106	0.251
	Violent discipline 1	−0.037	0.225	−0.100	0.020
	Dietary diversity 1	0.074	0.022	0.011	0.138
	Caregiver DERS 1	−0.058	0.078	−0.118	0.004
Communication	HOME Score 1	0.261	<0.001	0.196	0.325
	Violent discipline 1	0.037	0.193	−0.022	0.093
	Dietary diversity 1	0.032	0.288	−0.033	0.096
	Caregiver DERS 1	−0.033	0.291	−0.094	0.024
Problem solving	HOME Score 1	0.195	0.000	0.127	0.253
	Violent discipline 1	−0.015	0.600	−0.073	0.038
	Dietary diversity 1	0.068	0.021	0.011	0.125
	Caregiver DERS 1	−0.015	0.632	−0.075	0.043
Personal social	HOME Score 1	0.191	0.000	0.130	0.251
	Violent discipline 1	−0.027	0.328	−0.077	0.026
	Dietary diversity 1	0.079	0.009	0.020	0.140
	Caregiver DERS 1	−0.026	0.408	−0.089	0.037

Note: Numbers 0 refers to baseline, 1 refers to post-intervention and 2 refers to the 12-month follow-up variables. HOME = Home Observation for Measurement of the Environment; DERS = Difficulties in Emotion Regulation Scale. STDX = Standardized coefficient. 95% CI LB = Confidence Interval Lower Bound. 95% CI UB = Confidence Interval Upper Bound.

**Table 3:** Indirect effects of Sugira Muryango on child outcomes via caregiver behavior change.

Intervention	Caregiver Change	Child Outcome	STDX Coef.	*p*-Value	95% CI LB	95% CI UB
Child Gross motor						
Sugira Muryango	HOME	Gross motor	0.070	0.000	0.041	0.099
	Dietary Diversity	Gross motor	0.012	0.039	0.002	0.025
Child Communication						
Sugira Muryango	HOME	Communication	0.102	0.000	0.074	0.131
	Dietary Diversity	Communication	0.005	0.297	−0.005	0.018
Child Problem Solving						
Sugira Muryango	HOME	Problem-Solving	0.076	0.000	0.049	0.102
	Dietary diversity	Problem-Solving	0.011	0.047	0.001	0.024
Child Personal Social						
Sugira Muryango	HOME	Personal Social	0.075	0.000	0.050	0.100
	Dietary diversity	Personal Social	0.013	0.024	0.003	0.027

Note: HOME = Home Observation for Measurement of the Environment. STDX = Standardized coefficient. 95% CI LB = Confidence Interval Lower Bound. 95% CI UB = Confidence Interval Upper Bound.

**Table 4:** Total effects of Sugira Muryango on child outcomes.

Direct Effect		Indirect Effect	STDX Coef.	*p*-Value	95% CI LB	95% CI UB
Outcomes: Gross motor						
Treatment	+	via HOME	0.056	0.08	−0.012	0.237
	+	via dietary diversity	−0.002	0.954	−0.138	0.136
	+	via violent discipline	0.007	0.843	−0.12	0.143
	+	via DERS	−0.012	0.744	−0.155	0.117
Outcome: Communication						
Treatment	+	via HOME	0.075	0.016	0.026	0.264
	+	via dietary diversity	−0.024	0.471	−0.174	0.081
	+	via violent discipline	−0.035	0.277	−0.197	0.054
	+	via DERS	−0.059	0.074	−0.244	0.011
Outcomes: Problem-Solving						
Treatment	+	via HOME	0.073	0.022	0.021	0.266
	+	via dietary diversity	0.007	0.843	−0.12	0.143
	+	via violent discipline	−0.002	0.945	−0.132	0.124
	+	via DERS	−0.004	0.903	−0.14	0.121
Outcomes: Personal-Social						
Treatment	+	via HOME	0.015	0.62	−0.088	0.15
	+	via dietary diversity	−0.047	0.152	−0.219	0.034
	+	via violent discipline	−0.056	0.09	−0.239	0.018
	+	via DERS	−0.059	0.074	−0.244	0.011

STDX = Standardized coefficient. 95% CI LB = Confidence Interval Lower Bound. 95% CI UB = Confidence Interval Upper Bound. HOME = Home Observation for Measurement of the Environment; DERS = Difficulties in Emotion Regulation Scale. “+” indicate the combined direct and indirect effects as the total effect.

## Data Availability

Deidentified data from the original CRT are available in the World Bank data micro catalogue [https://microdata.worldbank.org/index.php/catalog/6107, (accessed on 17 February 2026)]. The analytic code necessary to reproduce the analyses presented in this paper is provided as Supplementary Material.

## Supplementary Materials

The following supporting information can be downloaded at: https://www.mdpi.com/article/10.3390/children13030344/s1. Table S1: Results from Structural Equation model that includes clustered estimates examined as a sensitivity analysis. Table S2: Alternative Model. Results from alternative structural equation model that included intermediary child development scores immediately post-intervention.
