# Supplementary material for: Mechanisms of Change Underlying Effects of an Early Parenting Intervention on Child Development Among Vulnerable Families in Rwanda
Source: Children (Basel). 2026 Feb 27;13(3):344. doi: 10.3390/children13030344 (PMC13025627; doi:10.3390/children13030344)
Supplement: Supplementary file 1 [file children-13-00344-s001.zip › children-4053120-supplementary.pdf]

SUPPLEMENTARY MATERIALS FOR :

Article

Mechanisms of change underlying effects of an early parenting intervention on child development among vulnerable families in Rwanda

Sarah K. G. Jensen [a], PhD ; Matias Placencio-Castro [b,c], PhD; Shauna M. Murray [d], MA; Vincent Sezibera [d], PhD ; Theresa S. Betancourt [b], ScD

[a]Women and Infants Hospital, Providence, MA, USA. ORCID: 0000-0001-6407-3055
[b]Boston College, School of Social Work, Chestnut Hill, MA, USA;
[c]Boston College, Lynch School of Education and Human Development, Chestnut Hill, USA;
[d]Boston University, Boston, USA.
[e]Center for Mental Health, University of Rwanda, Kigali, Rwanda
* Correspondence: **Author:** Theresa S. Betancourt; Phone: +1 617 890 9196; Fax: 617-552-8251; Email: theresa.betancourt@bc.edu

SUPPLEMENTAL Table 1. Results from Structural Equation model that includes clustered estimates examined as a sensitivity analysis.

Outcome	Predictor	STDX Coef.	P-value	95% CI LL	95% CI UL
**Auto-regressive path child development baseline to post-intervention**					
**Auto-regressive path child development baseline to 1-year follow-up**					
Gross motor 2	Gross Motor 0	0.156	0.000	0.006	0.013
Communication 2	Communication 0	0.140	0.000	0.005	0.010
Problem Solving 2	Problem Solving 0	0.144	0.000	0.005	0.012
Personal Social 2	Personal Social 0	0.172	0.000	0.008	0.015
**Auto-regressive path caregiver behavior baseline to post-intervention**					
HOME Score 1	HOME Score 0	0.283	0.000	0.044	0.065
Dietary diversity 1	Dietary diversity 0	0.252	0.000	0.147	0.238

Violent discipline 1	Violent discipline 0	0.507	0.000	0.275	0.375
Caregiver DERS 1	Caregiver DERS 0	0.517	0.000	0.024	0.030
**Treatment effects on post-intervention caregiver behavior**					
HOME Score 1	Treatment	0.390	0.000	0.677	0.893
Dietary div. 1	Treatment	0.169	0.000	0.221	0.454
Violent dis. 1	Treatment	-0.167	0.000	-0.440	-0.229
Caregiver DERS 1	Treatment	-0.054	0.043	-0.209	-0.004
**Treatment effects on endline child development outcomes**					
Gross Motor	Treatment	-0.015	0.678	-0.161	0.112
Communication	Treatment	-0.029	0.377	-0.184	0.069
Problem Solving	Treatment	-0.005	0.884	-0.142	0.120
Personal Social	Treatment	-0.061	0.066	-0.247	0.008
**Effects of post-intervention caregiver behaviors on endline child development outcomes**					
Gross motor	HOME Score 1	0.181	0.000	0.108	0.249
	Violent discipline 1	-0.037	0.261	-0.101	0.026
	Dietary diversity 1	0.075	0.020	0.012	0.139
	Caregiver DERS 1	-0.058	0.062	-0.117	0.003
Communication	HOME Score 1	0.266	0.000	0.198	0.323
	Violent discipline 1	0.038	0.196	-0.019	0.092
	Dietary diversity 1	0.032	0.299	-0.027	0.092
	Caregiver DERS 1	-0.034	0.271	-0.092	0.023
Problem solving	HOME Score 1	0.199	0.000	0.132	0.258
	Violent discipline 1	-0.016	0.615	-0.078	0.045
	Dietary diversity 1	0.069	0.022	0.009	0.125
	Caregiver DERS 1	-0.015	0.614	-0.074	0.044
Personal social	HOME Score 1	0.196	0.000	0.129	0.254
	Violent discipline 1	-0.027	0.337	-0.080	0.029
	Dietary diversity 1	0.081	0.010	0.021	0.139
	Caregiver DERS 1	-0.027	0.411	-0.090	0.036

Numbers 0 refers to baseline, 1 refers to post-intervention and 2 refers to the 12 months follow up. HOME = Home Observation for Measurement of the Environment; DERS = Difficulties in Emotion Regulation Scale. STDX = Standardized coefficient.

SUPPLEMENTAL Table 2. Alternative Model. Results from alternative structural equation model that included intermediary child development scores immediately post-intervention.

Outcome	Predictor	STDX Coef.	P-value	95% CI LL	95% CI UL
**Auto-regressive path child development baseline to post-intervention**					
Gross motor 1	Gross motor 0	0.303	0.000	0.014	0.021
Communication 1	Communication 0	0.484	0.000	0.023	0.028
Problem Solving 1	Problem Solving 0	0.226	0.000	0.010	0.016
Personal Social 1	Personal Social 0	0.203	0.000	0.010	0.017
**Auto-regressive path child development baseline to 1-year follow-up**					
Gross motor 2	Gross Motor 1	0.216	0.000	0.161	0.282
Communication 2	Communication 1	0.188	0.000	0.143	0.246
Problem Solving	Problem Solving	0.107	0.000	0.046	0.170
Personal Social	Personal Social	0.146	0.000	0.094	0.202
**Auto-regressive path caregiver behavior baseline to post-intervention**					
HOME Score 1	HOME Score 0	0.214	0.000	0.030	0.049
Dietary diversity 1	Dietary diversity 0	0.251	0.000	0.145	0.239
Violent discipline 1	Violent discipline 0	0.505	0.000	0.272	0.372
Caregiver DERS 1	Caregiver DERS 0	0.517	0.000	0.024	0.030
**Treatment effects on post-intervention caregiver behavior**					
HOME Score 1	Treatment	0.339	0.000	0.549	0.754
Dietary div. 1	Treatment	0.156	0.000	0.178	0.422
Violent dis. 1	Treatment	-0.170	0.000	-0.453	-0.233
Caregiver DERS 1	Treatment	-0.053	0.049	-0.218	0.000
**Treatment effects on endline child development outcomes**					
Gross Motor	Treatment	-0.023	0.518	-0.184	0.091
Communication	Treatment	-0.031	0.365	-0.189	0.073
Problem Solving	Treatment	-0.019	0.572	-0.160	0.090
Personal Social	Treatment	-0.072	0.033	-0.265	-0.013
**Effects of post-intervention caregiver behaviors on endline child development outcomes**					
Gross motor	HOME Score 1	0.121	0.001	0.051	0.193
	Violent discipline 1	-0.046	0.113	-0.101	0.005
	Dietary diversity 1	0.080	0.014	0.014	0.139

	Caregiver DERS 1	-0.055	0.064	-0.109	0.004
Communication	HOME Score 1	0.219	0.000	0.158	0.286
	Violent discipline 1	0.027	0.336	-0.025	0.077
	Dietary diversity 1	0.042	0.186	-0.021	0.102
	Caregiver DERS 1	-0.035	0.216	-0.089	0.017
Problem solving	HOME Score 1	0.148	0.000	0.080	0.217
	Violent discipline 1	-0.023	0.394	-0.075	0.032
	Dietary diversity 1	0.088	0.004	0.029	0.145
	Caregiver DERS 1	-0.021	0.528	-0.083	0.048
Personal social	HOME Score 1	0.151	0.000	0.080	0.217
	Violent discipline 1	-0.023	0.394	-0.075	0.032
	Dietary diversity 1	0.087	0.004	0.029	0.145
	Caregiver DERS 1	-0.021	0.528	-0.083	0.048

Numbers 0 refers to baseline, 1 refers to post-intervention and 2 refers to the 12 months follow up. HOME = Home Observation for Measurement of the Environment; DERS = Difficulties in Emotion Regulation Scale. STDX = Standardized coefficient.
